# Cyanogenic Compounds, Physicochemical Parameters and Metals in Water From Cassava Processing Areas: A Study in the Brazilian Amazon, Using Multivariate Analysis

**DOI:** 10.1002/tox.70067

**Published:** 2026-04-10

**Authors:** Alana Coêlho Maciel, Mariane Gama Nabiça, Luciana Pinheiro Santos, Thiago de Melo e Silva, Elivaldo Nunes Modesto Junior, Heronides Adonias Dantas Filho, Charles Miller de Souza Borges, Kelly das Graças Fernandes Dantas, Simone de Fátima Pinheiro Pereira, Ivan Carlos da Costa Barbosa, Beni Jequicene Mussengue Chaúque, Rosinelson da Silva Pena, Nayara Macêdo Peixoto Araújo, Alessandra Santos Lopes

**Affiliations:** ^1^ Postgraduate Program in Food Science and Technology, Technological Institute Federal University of Pará (UFPA) Belém Pará Brazil; ^2^ Laboratory of Applied Analytical Spectrometry, Institute of Exact and Natural Sciences Federal University of Pará (UFPA) Belém Pará Brazil; ^3^ Socioenvironmental and Water Resources Institute Federal Rural University of the Amazon (UFRA) Belém Pará Brazil; ^4^ Environmental Analytical Chemistry Laboratory Federal University of Pará (PPGQ) Belém Pará Brazil; ^5^ Institute of Exact and Natural Sciences Federal University of Pará (UFPA) Belém Pará Brazil; ^6^ Postgraduate Program in Chemistry Federal University of Pará (UFPA) Belém Pará Brazil; ^7^ Socio‐Environmental and Water Resources Institute Federal Rural University of the Amazon Belém Pará Brazil; ^8^ Center of Studies in Science and Technology (NECET), Biology Course Rovuma University, Niassa Branch Lichinga Mozambique; ^9^ Graduate Program in Food Science and Technology, Technological Institute Federal University of Pará (UFPA) Belém Pará Brazil

**Keywords:** Brazilian Amazon, cassava processing effluents, cyanogenic compounds, multi‐elements, physicochemical parameters

## Abstract

This study evaluated the water quality of artesian wells and effluents generated in cassava processing in a rural area of Bragança, Pará, with emphasis on physicochemical parameters, multielement composition, and cyanogenic compounds. The well water presented specific nonconformities, highlighting pH values below the recommended level and high concentrations of residual chlorine. Although cyanide was not detected in the groundwater, the effluents presented concentrations of total and free cyanide above legal limits, mainly in the pressing water and in the washing and fermentation tanks, indicating an environmental risk associated with the inadequate disposal of these residues. Regarding the multielement analysis, boron and copper enrichment was observed in the well water, and high concentrations of manganese and nickel were found in the effluents, exceeding the limits established by Brazilian environmental legislation. The multivariate analysis revealed distinct physicochemical profiles between the well water and the effluents, with temperature, dissolved oxygen, and pH being the main factors responsible for the differentiation between the matrices. The results indicate the need for improvements in the management and treatment systems of cassava processing effluents, aiming at protecting water resources and reducing potential environmental and human health risks.

## 1 Introduction

Cassava ( *Manihot esculenta* Crantz) is a crop of significant socioeconomic, cultural, and nutraceutical importance. It is extensively cultivated in tropical and subtropical regions worldwide [1]. In 2021, the world's largest cassava producers, in terms of tonnage, were Nigeria (63 031 377 t), the Democratic Republic of Congo (45 673 454 t), Thailand (30 108 352 t), Ghana (22 681 510 t), and Brazil, which ranked fifth with 18 098 115 tons [2]. By 2023, Brazil's production increased to 18 514 317 tons, with the North region contributing significantly (5 786 324 t). The state of Pará led production within this region (3 769 677 t), with the municipality of Acará standing out as the state's largest producer, yielding 326 tons [3].

In Pará, the high cassava production is complemented by intense artisanal processing of derivative products, including flour, *tapioca, tucupi, maniçoba, tarubá, caissuma*, and *tiquira*. However, this traditional production generates substantial volumes of effluents rich in cyanide, as well as various organic (e.g., starch, volatile compounds, fibers, cellulose, oils, fats, and phenolic compounds) and inorganic elements (e.g., mineral salts, nutrients, solids, and metals) [4, 5].

The improper and untreated disposal of liquid waste from cassava processing poses serious threats to environmental, animal, and human health. In Brazil, there is not much research that addresses this topic in depth. Only in Nigeria has a similar study been conducted, investigating levels of heavy metal contamination in soils exposed to raw and recycled effluents from cassava processing and their potential impact on soil and environmental health [6]. This is a knowledge gap for the scientific community and for rural populations in the Brazilian Amazon, who use cassava as a means of subsistence and income. This waste degrades water bodies by diminishing their self‐purification capacity, reducing dissolved oxygen levels, altering pH, and intensifying the eutrophication process in rivers, streams, and wells within affected areas. Furthermore, the exposure to and accumulation of heavy metals and cyanogenic compounds in these environments presents severe health risks, including reproductive disorders, neurotoxicity, cardiotoxicity, teratogenesis, mutagenesis, immunological disturbances, and carcinogenicity [1]. Given this, the hypothesis raised is that during the processing of cassava flour, at the stage of liquid waste disposal, contaminants are released into the soil and groundwater that exceed legal limits and pose an ecotoxicological and health risk to the rural community of Bragança in Pará.

To address these challenges, it is crucial to evaluate physicochemical parameters, trace elements, and contaminants to ensure the safety and quality of water sources, particularly wells. Additionally, implementing efficient effluent management practices during cassava processing, aligned with waste disposal regulations, is essential for mitigating these impacts.

This study aimed to assess the physicochemical properties, trace elements, and cyanogenic compounds in well water and cassava processing effluents in the rural area of Bragança, Pará.

## 2 Materials and Methods

### 2.1 Study Area

The study was conducted on a rural property located at 1°02′06.6″S, 46°49′42.8″W, on the outskirts of Bragança, in the Northeast Pará Mesoregion (Figure 1). The research was conducted on the property as a pilot project and selected based on specific criteria: it produces artisanal cassava flour, has a Geographical Indication (GI) seal certified by the Pará Agricultural Defense Agency (Adepará), and has the Family Farming seal, which guarantees the origin, quality, and traceability of the cassava production chain.

**FIGURE 1:**
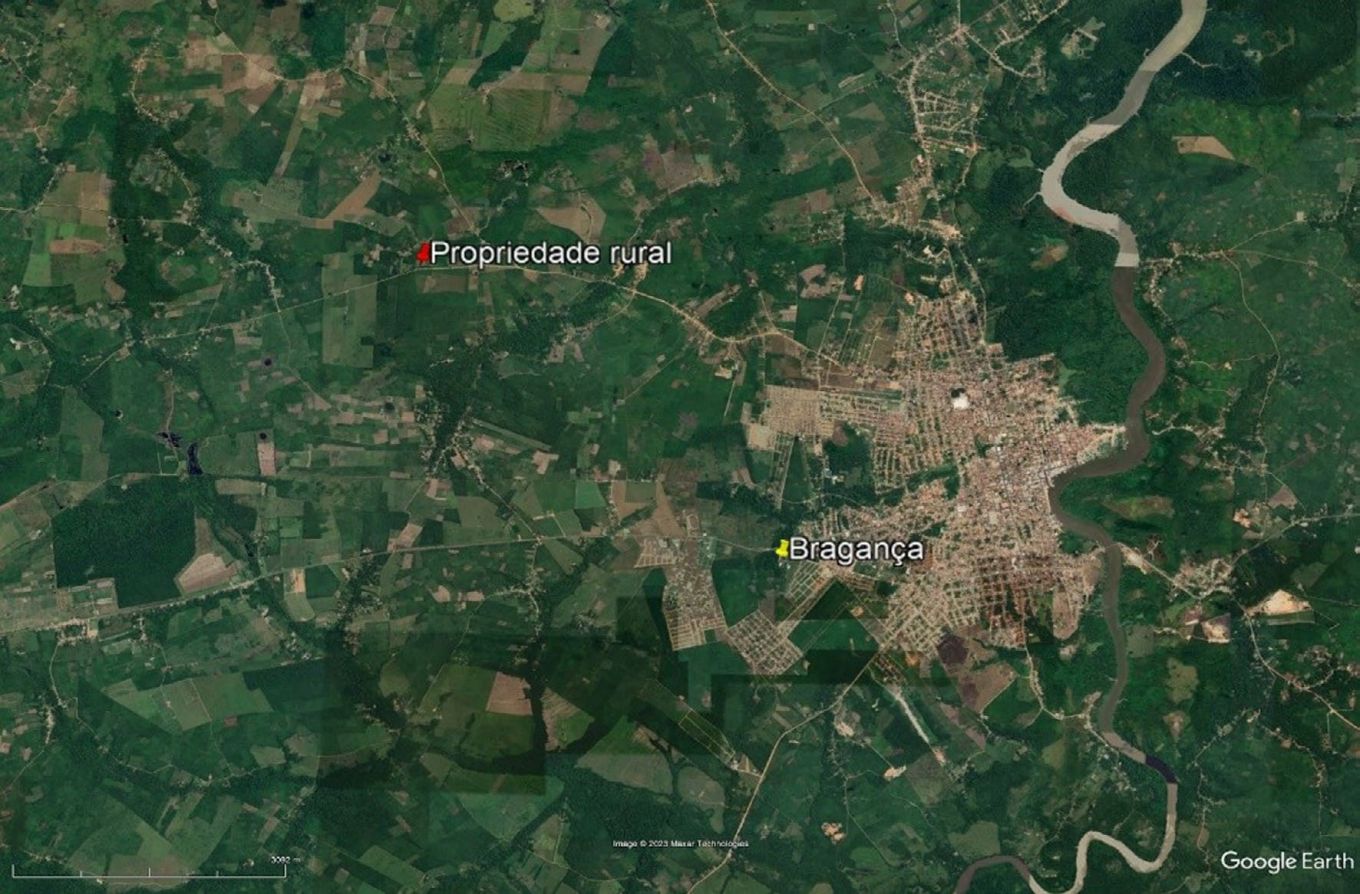
Location map of the study area on the rural property in the municipality of Bragança/PA.

### 2.2 Sample Collection

Water samples were collected in triplicate from two primary locations: an artesian well (Figure 2A) and an effluent pond (Figure 2B). Additional triplicate samples were taken from three points within the artisanal cassava processing environment: the fermentation tank (FT) (Figure 2C), the washing tank (WT) (Figure 2D), and the pressing water (WP) (Figure 2E), locally known as *manipueira*. The sampling was conducted during the amazonian winter (March 2023). Samples were stored in clean, sterile 500 mL polyethylene bottles, properly labeled, acidified to 2%, and transported to the laboratory in a thermal box to preserve their integrity.

**FIGURE 2:**
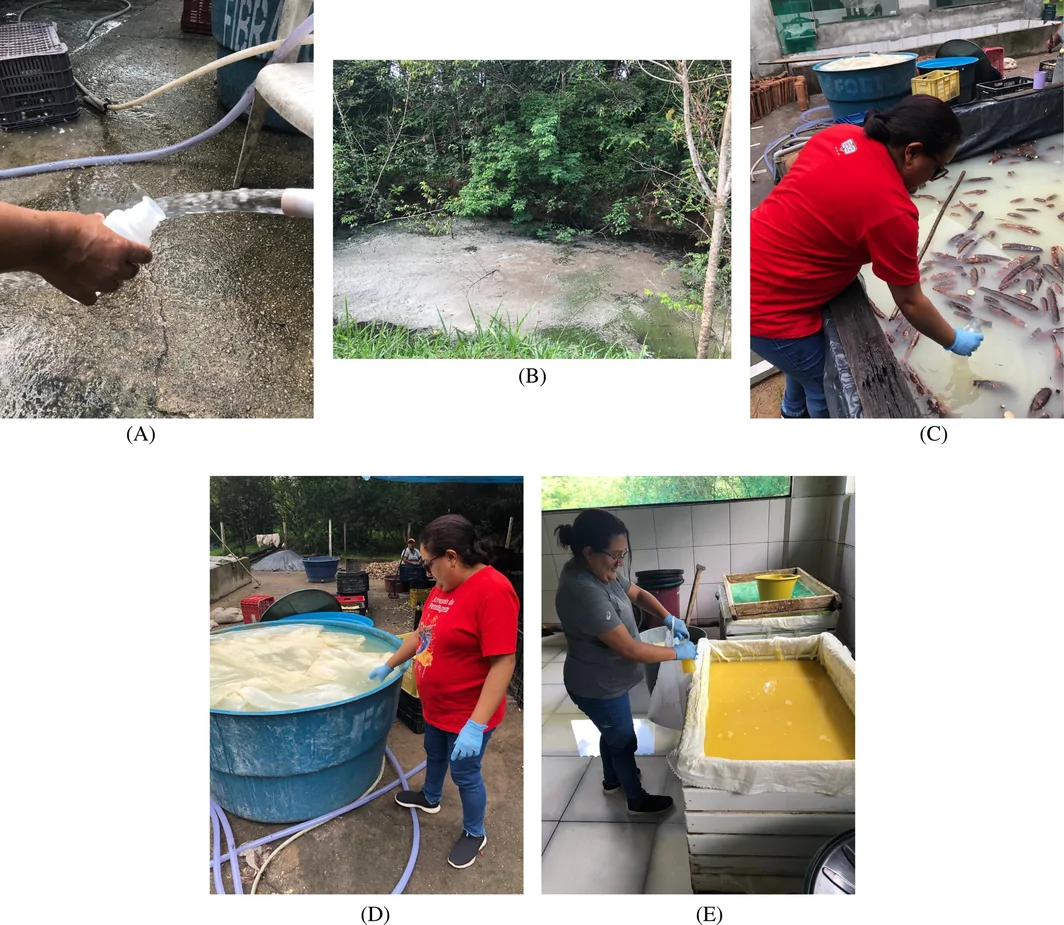
Sampling locations in water sources: (A) artesian well, (B) effluent pond, and in the artisanal cassava processing environment (C) fermentation tank, (D) washing tank, and (E) pressing water (manipueira).

### 2.3 Physicochemical Analysis

The analyses of temperature, alkalinity, acidity, chloride, total dissolved solids, total hardness, electrical conductivity, free residual chlorine, and dissolved oxygen were conducted at the Laboratory of Physical–Chemical Analysis, part of the Center for Agricultural Technology (CTA) at the Federal Rural University of the Amazon (UFRA). Turbidity, pH, Oxidation/Reduction Potential (ORP), and nitrate analyses were performed at the Laboratory of Analytical and Environmental Chemistry (LAQUANAM) at the Federal University of Pará (UFPA). Total and free cyanide analyses were carried out at the Laboratory of Amylaceous Sources (LAFAMI), also at UFPA. The analytical procedures and equipment used are detailed in Table 1.

**TABLE 1:** Parameters, methods and references used in water and effluent analyses.

Parameter	Unit	Determination	References
Turbidity	NTU	Turbidity meter—Instrutherm TD 300	—
Temperature	°C	KKcare Professional 5‐in‐1 multiparameter combined test meter PH/EC/TDS/salinity/thermometer	—
Hydrogen potential	—	pH Meter—Instrutherm—PH 710	—
Oxidation/reduction potential (ORP)	mV	ORP Meter Gondo Ezdo PCT 407	—
Alkalinity	mg•L^−1^	Method potentiometric titration	[7]
Acidity	mg•L^−1^		
Chloride	mg•L^−1^		
Total hardness	mg•L^−1^		
Nitrate		Spectrophotometric method with color development	[8]
Electrical conductivity	μS.cm^−1^	Conductivity Meter—Instrutherm—CD 850	—
Total dissolved solids	mg•L^−1^	KKcare Professional 5‐in‐1 multiparameter combined test meter PH/EC/TDS/salinity/thermometer	—
Free residual chlorine	mg•L^−1^	Chlorine Tester Akso—Digital portable chlorine meter	—
Dissolved oxygen	mg•L^−1^	Dissolved Oxygen Meter—Homis 8403 AZ	—
Total cyanide	mg•L^−1^	Bel UV‐M51 UV–Visible Spectrophotometer—190–1000 nm	[8]
Free cyanide	mg•L^−1^		

### 2.4 Trace Element Analyzes

Trace element analyses were conducted using an Inductively Coupled Plasma Optical Emission Spectrometer (ICP‐OES) (model iCAP 6500, Thermo Fisher Scientific, Cambridge, England) at the Applied Analytical Spectrometry Laboratory (GEAAp) of the Federal University of Pará (UFPA). High‐purity argon gas (99.999%) supplied by White Martins (Icoaraci, PA, Brazil) was used to purge the optics and sustain the plasma. The operational parameters used in ICP‐OES to perform the analyses are detailed in Table 2.

**TABLE 2:** Instrumental conditions for determining elements by ICP‐OES.

Parameters (ICP‐OES)	Valor
Nebulizer	Concentric
Misting chamber	Cyclonic
Radio frequency power (kW)	1.15
Plasma gas flow (L min^−1^)	12
Auxiliary gas flow (L min^−1^)	0.5
Nebulizer gas flow (L min^−1^)	0.5
Reading time(s)	10
Number of replicates	3
Element/λ (nm)	Ag 3280.068; Al 3082.15; As I 189.042; B 2496.678; Ba II 232.527; Cd II 214.438; Co 228.616; Cu II 224.700; Cr 283.563; Fe 238.204; K 769.896; Mn 257.610; Ni 221.647; Pb 216.999; Se 203.985; Zn 202.548

#### 2.4.1 Reagents and Solutions

Glassware and polyethylene bottles were decontaminated by submerging them in a 10% (v/v) nitric acid (HNO_3_) solution for 24 h, followed by thorough rinsing with deionized water. All reagents used were of analytical grade, and dilutions were prepared using ultrapure water generated by a Sinergy‐UV purification system (resistivity 18.2 MΩ·cm; Millipore, Bedford, USA).

To prepare analytical solutions and acidify water samples, 65% (v/v) nitric acid (Alphatec, Brazil) was purified using a sub‐distillation system (Bergof, model BSP 929‐IV, Germany). A multielement standard (Fluka Analytical, St. Gallen, Switzerland) compliant with ISO/IEC 17025 and ISO Guide 34 was used. It contained 1000 mg·L^−1^ (Specsol, São Paulo, Brazil) in 10% (v/v) nitric acid with the following concentration ranges: 1.0–5.0 mg·L^−1^ for Ag, Ba, Cd Co, Cu, Fe, Mn, and Zn; 5.0–25 mg·L^−1^ for Al, B, Cr, K, and Ni; and 10–50 mg·L^−1^ for Pb.

In addition, a monoelement standard for AA/ICP (Specsol, Brazil) containing 1000 mg·L^−1^ was used for Se (1.0–5.0 mg·L^−1^), and a standard for ICP (Fluka Analytical, St. Gallen, Switzerland) containing 250 mg·L^−1^ was used for As (1.0–5.0 mg·L^−1^).

#### 2.4.2 Sample Preparation and Analysis

Sample analysis followed previously established methods described in the literature (Pereira Junior and Dantas, 2016), with adaptations. In the laboratory, the samples were centrifuged, filtered, and their acidity was measured. Subsequently, triplicate samples (*n* = 3) were transferred to 14 mL vials, acidified with 1.4 mL of nitric acid (HNO_3_) to achieve a final acidity of 5%, and analyzed using ICP‐OES.

#### 2.4.3 Limit of Detection (LOD) and Limit of Quantification (LOQ)

The Limits of Detection (LOD) and Limits of Quantification (LQ) for ICP‐OES were calculated based on 10 readings of the analytical blank, as suggested by Thomsen, Roberts, and Burgess (2000), using the following equations:

(1) $$\mathrm{LD}=\left(3\times \mathrm{BEC}\times {\mathrm{RSD}}_{\text{Blank}}\right)/100$$

(2) $$\mathrm{LQ}=\left(10\times \mathrm{BEC}\times {\mathrm{RSD}}_{\text{Blank}}\right)/100$$

(3) $$\mathrm{SBR}=I_{\mathrm{SR}}–I_{\text{Blank}}/I_{\text{Blank}}$$

(4) $$\mathrm{BEC}=C_{\mathrm{SR}}/\mathrm{SBR}$$

where CSR represents the concentration of the multielement reference solution used in the calibration curve; SBR is the ratio of the analytical signal to the background signal; ISR and IBlank are the intensities of the reference solution and the blank signal (*n* = 10), respectively. RSDBlank refers to the relative standard deviation of the blank (*n* = 10).

### 2.5 Statistical Analysis

Descriptive statistics (mean, standard deviation, coefficient of variation, maximum, and minimum) were calculated for the physical–chemical parameters and potentially toxic metals using Microsoft Excel software. The Shapiro–Wilk test, conducted with Statistica 8.1, was used to assess data normality and determine the significance between water and effluent samples (independent groups). The variables turbidity, temperature, pH, ORP, alkalinity, acidity, chloride, hardness, nitrate, conductivity, total solids, residual chlorine, and dissolved oxygen followed a normal distribution (parametric), and the Student's *t*‐test was used to compare means. The Mann–Whitney test was applied to variables with a non‐normal distribution, including total cyanide, free cyanide, and potentially toxic metals (non‐parametric). A multiple correlation analysis was then performed using the Spearman test to evaluate correlations between the physical–chemical data (parametric) and potentially toxic metals (non‐parametric) in the water and effluent samples. Multivariate Analysis was employed to better extract data information using language R.

## 3 Results and Discussion

### 3.1 Physical–Chemical Parameters

The results of the analysis of the physical–chemical parameters of both the artesian well water and the effluents, along with the maximum permissible values according to Brazilian legislation, are presented in Table 3.

**TABLE 3:** Results of descriptive analyses of physical–chemical parameters in water and effluent samples compared to Brazilian legislation.

Parameters	Effluent pond (EL)				Artesian well (WA)				357/05^a^	430/11^b^	888/21^c^
	Average ± SD	CV %	Max	Min	Average ± SD	CV %	Max	Min			
Turbidity (NTU)	151.60 ± 1.18	0.78	153.00	150.00	0.18 ± 0.01	5.93	0.19	0.16	100	—	5.0
Temperature (°C)	27.84 ± 0.06	0.23	27.90	27.70	25.97 ± 1.04	4.00	27.10	23.08	—	≤ 40°C^d^	—
pH	6.07 ± 0.06	0.91	6.15	6.01	5.04 ± 0.05	1.08	5.21	4.99	6.0–9.0	5.0–9.0	6.0–9.5
ORP (mV)	85.77 ± 0.57	0.66	86.90	85.00	148.21 ± 0.13	0.09	148.40	148.00	—	—	—
Alkalinity (mg•L^−1^)	594.47 ± 0.83	0.14	596.00	594.00	4.41 ± 0.50	11.25	4.99	3.94	—	—	—
Acidity (mg•L^−1^)	209.07 ± 0.51	0.25	209.95	208.52	24.40 ± 0.42	1.73	24.74	23.70	—	—	—
Chloride (mg•L^−1^)	1397.57 ± 4.12	0.30	1399.60	1389.60	16.33 ± 0.56	3.40	16.99	15.50	250	—	250
Total hardness (mg•L^−1^)	890.33 ± 1.18	0.13	892.00	888.00	16.76 ± 1.02	6.06	17.97	15.01	—	—	300
Nitrate (mg•L^−1^)	0.17 ± 0.09	52.53	0.23	0.05	1.88 ± 0.28	14.76	2.32	1.03	10.0	—	10
Conductivity (μS cm^−1^)	198.13 ± 0.64	0.32	199.00	197.00	36.98 ± 0.59	1.58	37.90	36.22	—	—	—
Total solids (mg•L^−1^)	312.86 ± 0.43	0.14	314.12	312.40	24.07 ± 0.70	2.92	25.00	23.00	500	20%^e^	500
Free residual chlorine (mg•L^−1^)	0.00 ± 0.00	0.00	0.00	0.00	0.25 ± 0.25	99.17	1.16	0.13	0.01	—	5
Dissolved oxygen (mg•L^−1^)	3.90 ± 1.61	41.16	6.10	2.72	4.37 ± 0.18	4.07	4.87	4.09	≥ 6	—	—

^a^ Maximum value allowed for fresh waters—class 1.

^b^ Maximum values allowed for releasing effluents into a receiving body.

^c^ It should not exceed 3°C at the limit of the mixing zone.

^d^ Total suspended solids: minimum removal efficiency of 20%, after desanding.

The average turbidity of the artesian well water was 0.18 NTU, in compliance with the standards established by the legislation. In contrast, the average turbidity value of the effluent lagoon was 151.60 NTU. This high turbidity is largely due to the significant presence of total suspended solids (312.86 mg•L^−1^), including organic matter (such as algae, bacteria, and plankton) and inorganic particles (like sand and clay), which hinder the efficiency of the photosynthetic process in the aquatic environment [9, 10].

Water temperature is a crucial parameter in biological processes. The average temperature of the artesian well water was 25.97°C (ranging from 23.08°C to 27.10°C), while the effluent lagoon had an average of 27.84°C (ranging from 27.70°C to 27.90°C). The small thermal variation in the effluent lagoon suggests stable conditions. As temperature increases, the rates of chemical (gas transfer) and biological reactions rise, while the solubility of contaminants decreases and the solubility of oxygen also declines. In warmer water, solids dissolve more readily than in colder temperatures [10, 11].

The average pH of the artesian well water was 5.04, ranging from 4.99 to 5.21, which is below the range recommended by legislation (6.0–9.5), which are ideal for the protection of aquatic life. The pH influences the solubility, distribution (both free and ionized), and toxicity of several elements, including the precipitation of metals [12, 13]. For the effluents, the average pH was 6.07 ± 0.06, in accordance with the legislative guidelines.

Although the oxidation–reduction potential (ORP) is not specified in legislations, it is an important indicator of the bacteriological quality of water, such as indicating the death of *Escherichia* *coli* when the ORP value is negative and equal to or greater than 650 mV. Although the pH was within the established limits, the average positive ORP value in the artesian well water was 148.21 mV, suggesting favorable conditions for the growth of bacteria [14]. In the effluents, the average positive ORP was 85.77 mV, indicating that the availability of elements such as carbon, hydrogen, oxygen, and nitrogen is lower, as these are consumed by the presence of an oxidizing agent (substances and/or organic matter), leaving anaerobic bacteria [15, 16].

Alkalinity plays a key role in neutralizing acids in natural waters, primarily through compounds such as carbonic acid, carbonates $\left(CO_{3}^{2-}\right)$, bicarbonates $\left(\mathrm{HC}O_{3}^{-}\right)$, hydroxides (OH^−^), and ions from calcium, magnesium, silicates, borates, phosphates, and ammonia. It is influenced by factors like climate, morphology, and human activities [17, 18]. Alkalinity is closely associated with hardness and is conditioned by pH, as higher alkalinity values lead to more stable pH levels, which in turn support physical, chemical, and biological processes in aquatic environments. In this study, the average alkalinity was 4.41 mg·L^−1^ in the water and 594.47 mg·L^−1^ in the effluents. This parameter is not defined in Brazilian legislation (No. 357/05 and No. 888/21) [19, 20].

While alkalinity does not pose a direct health risk, it can cause water rejection due to taste changes. However, exceptions exist, particularly when alkalinity is caused by hydroxides or contributes to increased total solids. The high alkalinity observed in the effluents may be linked to the trophic conditions in the lagoon, which influence CO_2_ variations and promote increased alkalinity ([17, 18, 21]) (Miranda et al. 2009).

Acidity refers to the water's ability to react with a strong base, influenced by the presence of strong mineral acids, weak acids (such as carbonic and acetic acids), and salts (like iron or aluminum sulfates), at a specific pH value [16]. Acidity is not defined in water potability regulations for water, natural waters, or effluents [19, 20, 22]. However, the average values found were 24.40 mg·L^−1^ for water and 209.07 mg·L^−1^ for effluents. Acidity plays a significant role in corrosion, speciation, chemical reaction rates, and biological processes [16].

The chloride ion, originating from both anthropogenic and geological sources, is one of the primary inorganic anions found in water and effluents. Its concentration influences the sensory properties of water: when sodium is the predominant cation, a salty taste may occur at concentrations of ≥ 250 mg·L^−1^, whereas water with calcium and magnesium as the primary cations exhibits no detectable taste even at concentrations up to 1000 mg·L^−1^ [16]. Elevated chloride levels in freshwater can lead to salinization, soil degradation, inhibited plant growth, and toxicity to aquatic organisms, including fish and invertebrates. Additionally, chloride interferes with the osmotic processes of aquatic species ([23, 24]). In freshwater and wastewater, chloride ions (Cl^−^) can form metal chlorides (e.g., CuCl and AgCl), metal oxychlorides (e.g., BiOCl), and precipitates such as Friedel's salt [Ca_2_Al(OH)_6_Cl·2H_2_O or Ca_4_Al_2_Cl_2_(OH)_12_] [23, 24]. In effluents, high chloride concentrations increase corrosivity and inhibit microbial activity by damaging cell membranes, disrupting metabolic pathways, and destabilizing the intracellular environment, which can lead to cell death or reduced metabolic efficiency [25]. In this study, the chloride concentration in artesian well water averaged 16.33 mg·L^−1^, aligning with the standards outlined in legislations No. 357/05 and No. 888/21. In effluents, however, the average concentration was significantly higher at 1397.57 mg•L^−1^, a parameter not addressed in legislation No. 430/11.

In this study, the average chloride concentration in water from the artesian well was 16.33 mg•L^−1^, which complies with Brazilian legislation (n° 357/05 and n° 888/21). However, the effluents had a much higher average chloride concentration of 1397.57 mg•L^−1^. Notably, Brazilian legislation (n° 430/11) does not specify limit values for chloride ion concentrations.

The average total hardness of the artesian well water was 16.76 mg•L^−1^ in the form of CaCO_3_, therefore within the permitted limit of 300 mg·L^−1^ CaCO_3_ by Brazilian legislation. Conversely, the effluents exhibited an average hardness of 890.33 ± 1.18 mg·L^−1^ CaCO_3_, which is not regulated under Resolution n° 430/11. Total hardness reflects the combined concentrations of calcium and magnesium ions in water, often associated with sulfate ions [26]. To a lesser extent, it may also include metallic ions such as iron, aluminum, zinc, manganese, and strontium. Factors like pH and dissolved oxygen can further influence water hardness.

Water hardness can be categorized as temporary or permanent. Temporary hardness results from calcium and magnesium bicarbonates, which decompose into carbon dioxide, water, and insoluble carbonates upon heating, making it removable by boiling. Permanent hardness, on the other hand, arises from soluble calcium and magnesium sulfates, chlorides, and nitrates [16, 18]. In Brazil, water is classified as hard when its hardness exceeds 150 mg•L^−1^, moderately hard between 75 and 150 mg•L^−1^, and soft when below 75 mg•L^−1^.

A study by Yousefi et al. [27] highlighted a significant relationship between water hardness and hypertension prevalence. Regions with low water hardness (46.84%; 95% CI: 37.61–57.64) had a higher prevalence of hypertension and pre‐hypertension compared to those with high water hardness (23.15%; 95% CI: 18.11–29.15). Moreover, age, body mass index (BMI), and waist circumference were strongly associated with hypertension.

The average nitrate concentration in the artesian well water was 1.88 mg·L^−1^, therefore below the limits established for Class 1 freshwater and potable water standards for human consumption. In contrast, the nitrate concentration in the effluents was 0.17 mg·L^−1^, for which no limit is specified in Resolution n° 430/2011. Excessive nitrate consumption, whether from contaminated water or food (such as processed, smoked, or grilled products, dairy, and vegetables), can pose significant health risks. Once ingested, nitrate is converted into nitrite by commensal bacteria in the mouth and gastrointestinal tract. Nitrite can then form nitrosamines—carcinogenic compounds. High nitrate concentrations in well water are particularly harmful to health, posing an increased risk for children and adults [28, 29, 30, 31].

Conductivity, a measure of the water's ability to conduct electrical current, reflects the concentration of dissolved ions such as aluminum, calcium, chloride, iron, magnesium, nitrate, phosphate, sodium, and sulfate, and is influenced by temperature [16]. Although this parameter is not regulated by current legislation, the artesian well water exhibited an average conductivity of 36.98 μS cm^−1^, while the effluents showed a higher conductivity of 198.13 μS·cm^−1^. In water with an ideal pH range of 6.0 to 9.0 [22] and high conductivity, the presence of dissolved salts generally improves its suitability for consumption. However, this was not observed in the artesian well water, where the pH (5.04) and conductivity were lower compared to the effluents, which had a pH of 6.07 and higher conductivity. This discrepancy suggests the presence of pollutants in the effluents [12].

The total solids (TS) concentrations in both the artesian well water (24.07 ± 0.70 mg·L^−1^) and the effluents (312.86 ± 0.43 mg•L^−1^) were within the limits established by current legislation. Total solids consist of suspended solids (TSS), such as visible particles like sand, clay, organic waste, or silt, and dissolved solids (TDS), which include salts, minerals, metals, and organic compounds. High concentrations of dissolved solids, comprising both organic and inorganic ions (e.g., carbonate, bicarbonate, chloride, sulfate, phosphate, nitrate, calcium, magnesium, and sodium), can harm aquatic ecosystems. Several factors can influence total solids concentration, such as soil erosion (caused by rain or wind), biological activity (e.g., organic matter or algae growth), and anthropogenic sources, including agricultural activities (fertilizers and pesticides) or industrial effluent discharge.

The average free residual chlorine (FRC) in the artesian well water was 0.25 ± 0.25 mg•L^−1^, ranging from 1.16 to 0.13 mg•L^−1^. This exceeds the limit established by current legislation (0.01 mg•L^−1^). FRC exists in water in the forms of hypochlorous acid (HClO) and hypochlorite ion (ClO^−^), as described by reactions 1 and 2 [32, 33].

(5) $${\mathrm{Cl}}_{2}+H_{2}O\to {\text{HOCl}}^{-}+H^{+}+{\mathrm{Cl}}^{-}\;\left(\text{hydrolysis process}\right)$$

(6) $${\text{HOCl}}^{-}\to H^{+}+{\mathrm{OCl}}^{-}\;\left(\text{ionization process}\right)$$

Chlorine residual levels (CRL) in appropriate concentrations enhance water disinfection through antimicrobial action. However, high levels of hypochlorous acid (HClO) can lead to issues such as corrosion, unpleasant odors, and acute or chronic health effects, including damage to the eyes, skin, lungs, and respiratory system ([23, 24]). For effective disinfection, a water pH near 5 is optimal, as this favors the presence of HClO in its strongest form. At this pH, HClO can dissociate to form chlorine gas, facilitating its penetration into the cell walls and protective layers of pathogens. This action enhances its antimicrobial efficacy by oxidizing sulfhydryl groups in certain enzymes, thereby disrupting protein synthesis and decarboxylating amino acids into nitrites and aldehydes [34, 35]. Notably, no CRL was detected in the effluent samples.

Dissolved oxygen (DO) serves as a key indicator of organic pollution in water bodies [36]. The artesian well water had an average DO value of 4.37 ± 0.18 mg•L^−1^, ranging from 4.87 to 4.09 mg•L^−1^. This value falls below the minimum recommended limit of ≥ 6 mg•L^−1^ set by Brazilian legislation (Brazil, 2005). DO is slightly soluble in water, with its solubility influenced by factors such as pressure (altitude), temperature, and dissolved salts [37]. Its concentration also depends on physical, chemical, and biochemical activities [16].

The reduced DO levels in the artesian well water suggest a higher reducing tendency in the environment, driven by substantial organic matter contributions. Organic waste from livestock (pigs and cattle) and domestic activities likely percolates or infiltrates into the groundwater. This organic matter undergoes chemical and biochemical oxidation by microorganisms, consuming dissolved oxygen [38]. Additionally, elevated temperatures exacerbate the reduction of oxygen solubility in water bodies [10].

In the effluent samples, the average DO was 3.90 ± 1.61 mg•L^−1^. This low value can be mainly attributed to the intense decomposition of organic compounds received together with the substantial volume of liquid waste generated during the artisanal production of cassava flour. These processes deplete oxygen in the effluent lagoon (Passos et al. 2018).

A Spearman correlation analysis was conducted to evaluate the relationships between the physicochemical parameters, as summarized in Table 4.

**TABLE 4:** Correlation of physical–chemical parameters of artesian well water and effluent pond samples on rural property in the municipality of Bragança/PA.

Variables	R	Artesian well water (WA)—Interpretation
Turbidity/alkalinity	0.579955	Moderate, positive and directly proportional correlation. Increases in turbidity tend to be accompanied by increases in alkalinity.
Turbidity/nitrate	−0.57963	Moderate, negative and inversely proportional correlation. When turbidity increases, nitrate levels tend to decrease.
pH/alkalinity	−0.53895	Moderate/negative/inversely proportional.
ORP/chloride	−0.60401	Moderate/negative/inversely proportional.
Alkalinity/nitrate	−0.607023	Moderate/negative/inversely proportional.
Acidity/DO	0.562054	Moderate/positive/directly proportional.
Total solids/residual chlorine	−0.688863	Moderate, negative and inversely proportional correlation. As total solids increase, there is a decrease in residual chlorine.

Variables	R	Efluent pond (EL)—Interpretation
pH/chloride	0.64946	Moderate, positive, and directly proportional correlation. Increases in pH are moderately related to increases in chloride levels.
pH/nitrate	0.58041	Moderate/positive/directly proportional.
pH/DO	0.593127	Moderate, positive, and directly proportional correlation. Indicates that higher pH levels are related to higher dissolved oxygen.
ORP/nitrate	0.551545	Moderate/positive/directly proportional.
Chloride/conductivity	−0.534508	Moderate/negative/inversely proportional.
Nitrate/DO	0.917227	Strong, positive, and directly proportional correlation. Higher nitrate levels are strongly associated with increases in dissolved oxygen.

The strongest correlation was observed between nitrate and DO (*r* = 0.917), indicating a strong and direct linear relationship (Figure 3a–c). Among the moderate correlations, a negative relationship was found between TS and CRL (*r* = −0.688), demonstrating a decreasing trend. Additionally, turbidity and alkalinity showed a moderate positive correlation (*r* = 0.580), reflecting an upward trend with some dispersion around the trend line.

**FIGURE 3:**
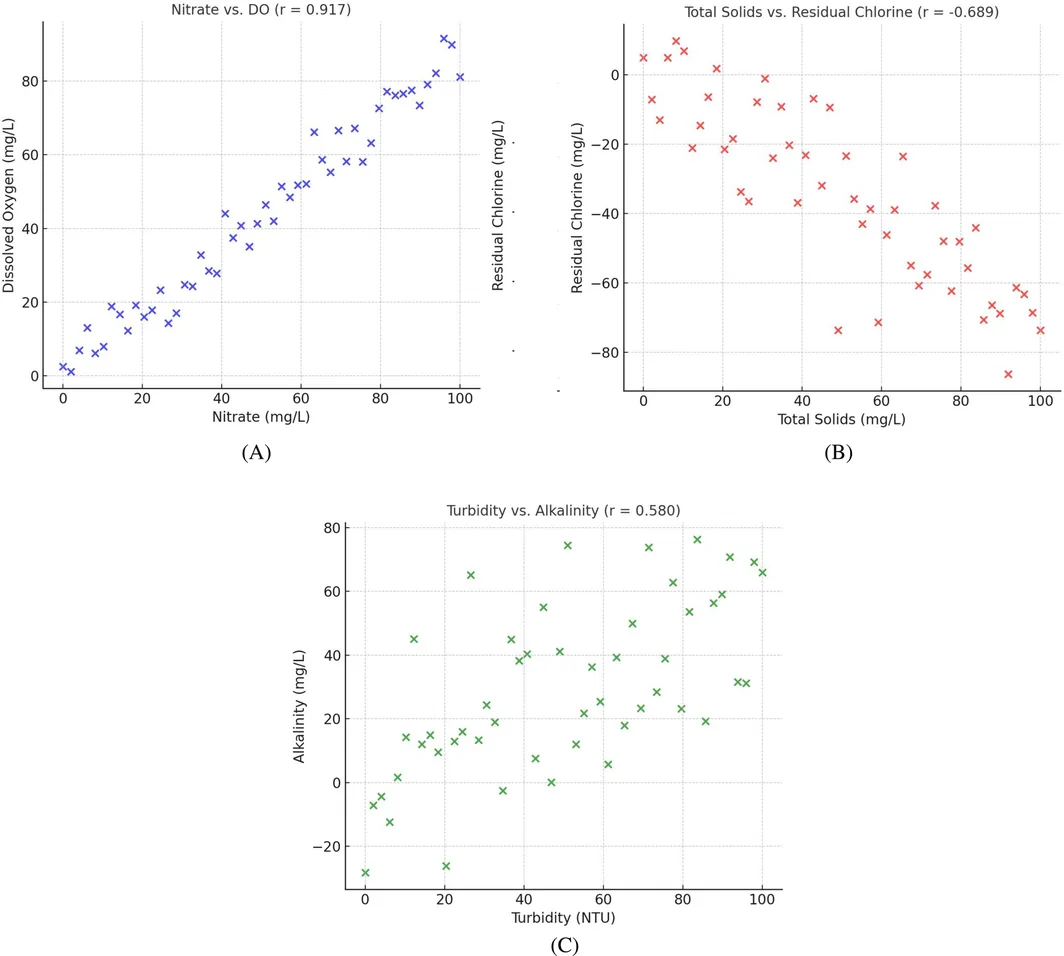
(A–C) Scatter plots for the three pairs of variables with the most significant correlations.

### 3.2 Total and Free Cyanide Levels

Total and free cyanide were not detected in the water samples from the artesian well. According to CONAMA legislation no. 357/05, the maximum allowable concentration of free cyanide is 0.005 mg•L^−1^, although it does not address total cyanide. Similarly, legislation no. 888/21 does not specify parameters for either total or free cyanide. The average concentrations of total and free cyanide at each stage of cassava processing, as well as in the generated waste, are summarized in Table 5.

**TABLE 5:** Results of total and free cyanide data present in the effluent stages generated.

Samples	Total cyanide [mg•L^−1^]					Free cyanide [mg•L^−1^]				
	Average ± SD	CV %	Max	Min	430/11	Average ± SD	CV %	Max	Min	430/11
Tank	1.66 ± 0.04	2.66	1.70	1.61	1.0	0.20 ± 0.02	12.43	0.22	0.17	0.2
Water tank	7.21 ± 0.48	0.07	7.71	6.75		0.14 ± 0.00	0.49	0.14	0.14	
Washing water (*manipueira*)	5.58 ± 0.33	0.06	5.83	5.21		0.79 ± 0.04	4.99	0.83	0.76	
Effluent pond	1.39 ± 0.37	0.26	1.71	0.99		0.07 ± 0.00	2.58	0.07	0.07	

The results revealed that the total cyanide concentrations in the effluents generated at all stages of cassava processing (tank, water tank, washing water, and effluent pond) exceeded the maximum limit permitted by law (1.0 mg•L^−1^). Regarding free cyanide, only the pressing stage recorded concentrations above the permissible limit at 0.79 mg•L^−1^.

High concentrations of cyanide are found in raw cassava residues, ranging from 114.7 to 367159.6 mg/kg, while cassava peels contain even higher concentrations, ranging from 360.05 to 368509.51 mg/kg in the soil [39]. Waste materials from cassava processing, including cassava remains, peels, fibers, and *manipueira* (liquid effluent rich in cyanogenic glycosides such as linamarin and lotaustralin), are often discarded or left to decompose. These wastes pose significant environmental risks by contaminating surface and groundwater, degrading soil quality, and negatively impacting plants and soil microbiota. Cyanogenic compounds can persist in the soil for extended periods, influenced by factors such as pH, organic matter content, cation exchange capacity (CEC), and clay composition. This persistence also facilitates the accumulation of toxic metals and further disrupts microbial communities [39, 40].

In this study, concentrations of the metals Ba (1.07 mg•L^−1^), Cu (0.56 mg•L^−1^), Mn (6.18 mg•L^−1^), and Ni (2.2 mg•L^−1^) were detected in effluents with pH levels ranging from 6.01 to 6.15. Cyanide in these conditions can form various metal‐cyanide complexes, including: Barium cyanide (Ba(CN)_2_)—highly soluble in water and decomposes under acidic conditions to release toxic hydrocyanic gas (HCN); dicyanocuprate ion (Cu(CN)_2_^−^) and copper cyanide (CuCN)—slightly soluble, prone to precipitating in soil, and more stable under alkaline conditions. Manganese cyanide (Mn(CN)_2_)—rare but possible in soils with low acidity; and tetracyanonichelate ([Ni(CN)_4_]^2−^)—which is highly stable and soluble, particularly in neutral to alkaline environments [41, 42].

Cyanide complexes with Ni and Cu tend to form weak bonds, adopting planar or square tetrahedral configurations. These complexes have relatively low binding energy, allowing them to dissociate easily and release free cyanide, which poses environmental risks. Cyanide complexes with Mn dominate across the pH range of 0–14, peaking in stability between pH 9 and 10 [41].

From a human health perspective, cyanide exposure can have systemic effects on multiple organ systems, including the respiratory, cardiovascular, gastrointestinal, hematological, endocrine, dermatological, musculoskeletal, hepatic, renal, and ocular systems. It can also affect body weight and neurological function, with severe cases leading to death [1, 42].

### 3.3 Total Contents in the Effluent Lagoon and Artesian Well

The atomic emission lines used for trace element determinations by ICP‐OES, using the parameters of limit of detection (LOD), limit of quantification (LOQ), and precision (via recovery assay), are presented in Tables 6 and 7, which followed the appropriate methods [43, 44]. The validation of the analytical method used for multielement determination by Inductively Coupled Plasma Optical Emission Spectrometry (ICP‐OES) was performed in accordance with the guidelines established by ANVISA (RE No. 899/2003) and INMETRO (DOQ‐CGCRE‐008/2010) [45], ensuring the reliability and traceability of the analytical results. The determination of the limits of detection (LOD) and limits of quantification (LOQ) is essential to evaluate the sensitivity and capability of the method to detect and quantify low analyte concentrations in complex matrices, guaranteeing that the measured values are distinguishable from background noise and exhibit acceptable levels of accuracy and precision. The LOD represents the lowest concentration that can be detected, though not necessarily quantified, whereas the LOQ corresponds to the minimum concentration that can be quantified with adequate accuracy and precision, defining the reliable working range of the method. Precision was assessed through the spike and recovery test, in which known concentrations of each analyte were added to the samples to determine the percentage recovery and to verify the absence of matrix interferences in the analytical response. Recovery rates ranged from 81% to 120%, in accordance with accepted validation criteria. These parameters confirm that the ICP‐OES method employed exhibits appropriate sensitivity, accuracy, and reproducibility for the quantification of metallic and trace elements in groundwater and cassava‐processing effluent samples.

**TABLE 6:** Limits of detection (LOD) and quantification (LOQ).

Element	SBR	C_SR_ [mg•L^−1^]	BEC [mg•L^−1^]	LOD [mg•L^−1^]	LOQ [mg•L^−1^]	*R* ^2^
Ag	65.56	5	0.08	0.002	0.008	0.9905
Al	55.77	25	0.45	0.011	0.037	0.9983
As	67.25	5	0.07	0.009	0.031	0.9970
B	131.6	25	0.19	0.015	0.050	0.7629
Ba	627.7	5	0.01	0.001	0.004	0.9986
Cd	2009	5	0.002	0.000	0.001	0.9984
Co	464.2	5	0.01	0.002	0.005	0.9986
Cu	368.4	5	0.01	0.002	0.007	0.9985
Cr	294.1	25	0.09	0.003	0.010	0.9987
Fe	20.57	5	0.24	0.029	0.096	0.9937
K	231.3	25	0.11	0.001	0.003	0.9913
Mn	210.6	5	0.02	0.001	0.004	0.9987
Ni	2834	25	0.01	0.001	0.004	0.9984
Pb	172.60	50	0.29	0.043	0.143	0.9980
Se	40.22	5	0.12	0.017	0.057	0.9974
Zn	1692	5	0.003	0.0004	0.001	0.9986

Abbreviations: BEC, background equivalent concentrations; CSR, concentration of the multielement reference solution; LOD, limits of detection; LOQ, limits of quantification; *R* ^2^, determination coefficient and type of fit linear; SBR, signal‐to‐background ratios.

**TABLE 7:** Assessment of the accuracy of the sample preparation procedure by the analyte addition and recovery method.

Element	Added (contamination)%	Recovered (white)%
Ag	1.5;2.5	109;114;114
Al	7.5;12.5	119;120;118
As	1.5;2.5;3	107;114;117
B	7.5;12.5	114;118;115
Ba	1.5;2.5	112;111;114
Cd	1.5;2.5	114;115;119
Co	1.5;2.5	109;110;112
Cu	1.5;2.5	120;120;112
Cr	7.5;12.5	118;120;110
Fe	1.5;2.5	114;103;112
K	17.3;22.5	81;102;118
Mn	1.5;2.5	91; 101;107
Ni	7.5;12.5	114;115;120
Pb	15;25	113;115;114
Se	1.5;2.5	82;102;114
Zn	1.5;2.5	116;115;116

The elements monitored in the artesian well water and effluent lagoon during this investigation include the concentrations (mg•L−1 mean ± SD, *n* = 3) shown in Table 8.

**TABLE 8:** Total concentration [mg•L^−1^] (mean ± standard deviation. *n* = 3) of Ag, Al, As, B, Ba, Cd, Co, Cu, Cr, Fe, K, Mn, Ni, Pb, Se and Zn in the effluent pond and well water with the respective maximum values allowed by Brazilian legislation.

Element	Effluent lagoon	Well water	430/11^a^	357/05^b^	888/21^c^
Ag	< 0.002	< 0.002	0.1	0.05	—
Al	< 0.011	< 0.011	—	0.1	0.2
As	< 0.009	< 0.009	0.05	0.01	0.01
B	< 0.015	1.23 ± 0.07	5.0	0.5	—
Ba	1.07 ± 0.06	0.20 ± 0.03	5.0	0.7	0.7
Cd	< 0.0004	< 0.0004	0.2	0.001	0.003
Co	< 0.002	< 0.002	—	0.05	—
Cu	0.56 ± 0.04	2.761 ± 0.004	1.0	0.009	2
Cr	< 0.003	< 0.003	0.1 (Cr^+6^)/1.0 (Cr^+3^)	0.05	0.05
Fe	< 0.029	< 0.029	15.0	0.3	0.3
K	< 0.001	1.2 ± 0.2	—	—	—
Mn	6.18 ± 0.035	0.48 ± 0.030	1.0	0.5	0.1
Ni	2.2 ± 0.2	< 0.001	2.0	0.025	0.07
Pb	< 0.043	< 0.043	0.5	0.033	0.01
Se	< 0.017	< 0.017	0.3	0.05	0.04
Zn	< 0.004	0.045 ± 0.002	5.0	5	5

^a^ [mg•L^−1^] maximum values allowed for releasing effluents into a receiving body.

^b^ [mg•L^−1^] maximum values allowed for fresh waters—class 1.

^c^ [mg•L^−1^] maximum permitted values for water for human consumption.

The concentrations of Ag, Al, As, B, Cd Co, Cr, Fe, K, Pb, Se, and Zn in the water from the effluent lagoon were below the detection limit (< LOD, Table 6). However, levels of Mn>Ni > Ba>Cu exceeded the detection limit. Among these, Ba (1.07 mg•L^−1^) and Cu (0.56 mg•L^−1^) complied with effluent discharge standards (Conama Resolution No. 430/11). Conversely, Mn (6.18 mg•L^−1^) and Ni (2.2 mg•L^−1^) exceeded the regulatory limits of 1.00 mg•L^−1^ and 2 mg•L^−1^, respectively.

In the artesian well water, Ag, Al, As, Cd Co, Cr, Fe, Ni, Pb, and Se were also below the detection limit (< LOD, Table 6). However, Cu > B > K > Mn > Ba > Zn were detected above the limit. Both B (1.23 mg•L^−1^) and Cu (2.761 mg•L^−1^) exceeded the freshwater quality standards set by Conama Resolution No. 357/05, which establish limits of 0.5 mg•L^−1^ for B and 0.009 mg•L^−1^ for Cu. Ba (0.20 mg•L^−1^), Mn (0.48 mg•L^−1^), and Zn (0.045 mg•L^−1^) were within acceptable levels under this resolution.

Heavy metals and trace elements can be termed as potentially hazardous elements (PHEs). While some of these elements are essential micronutrients at low concentrations, supporting enzymatic activities, they can become harmful to human health at higher levels.

According to the ATSDR [46], acute or chronic exposure to Ba can occur in the workplace or through the ingestion of contaminated food and water. Symptoms of exposure include gastrointestinal disorders, vomiting, abdominal cramps, diarrhea, difficulty breathing, changes in blood pressure, numbness around the face, muscle weakness, kidney damage, and in severe cases, changes in heart rhythm, paralysis, or death. Barium carbonates and sulfates remain in the environment for extended periods due to their poor solubility in water. In contrast, barium chlorides, nitrates, and hydroxides are more soluble in water but do not accumulate in the environment. The US Environmental Protection Agency (EPA) sets the maximum permissible barium concentration in drinking water at 2.0 mg•L^−1^, while Brazilian legislation (Resolutions 357/05 and 888/21) establishes a more stringent limit of 0.7 mg•L^−1^ [22, 46]. Barium in water can bioaccumulate in fish and plankton, with younger specimens accumulating up to 2.5 times more than adults, depending on factors such as tissue type, age, and environmental conditions [47].

Mn, another naturally occurring element, is essential for human health, being present in air, soil, and water. At low concentrations, it provides health benefits, but at higher levels, it can be toxic. Manganese can be released into water in both dissolved and suspended forms, depending on factors like pH, temperature, the presence of anions, and oxidation–reduction potential. It also accumulates in sediment and soil, where it does not decompose but rather changes its form, binding or separating from particles depending on the environment's conditions, such as cation exchange capacity (CEC) and organic matter content [48]. The EPA has set the maximum limit for manganese in drinking water at 1 mg•L^−1^, in bottled water at 0.05 mg•L^−1^, and in air at 5 mg m^−3^ [49]. Manganese primarily exists in the divalent form (Mn^2+^) between pH 4 and 7, and at higher pH levels, more oxidized forms emerge, which can oxidize microorganisms [48]. Manganese bioaccumulates in lower organisms such as phytoplankton, algae, mollusks, and certain fish, but not in higher organisms [48, 49]. Exposure to manganese occurs through ingestion of contaminated food and water, occupational exposure, and environmental disasters, such as the iron extraction mine disaster at Córrego do Feijão in Brumadinho, Brazil. Long‐term exposure can affect the nervous, pulmonary, and reproductive systems and may increase the risk of cancer [49, 50].

B is an essential element for plant nutrition; however, its ecotoxicity can inhibit plant growth, particularly in arid and semiarid regions, with contamination often resulting from the use of insecticides and fertilizers [51]. In humans, B at appropriate concentrations aids in the absorption of Ca, Mg, and vitamin D, contributing to bone health, wound healing, and the regulation of estrogen and testosterone levels. It also reduces inflammatory biomarkers, enhances antioxidant enzyme levels, protects against oxidative stress caused by pesticides and heavy metal toxicity, improves cognitive performance and memory, and supports the electrical activity of the brain. Additionally, B has preventive and therapeutic effects against several cancers, including prostate, cervical, lung, and non‐Hodgkin lymphoma [52].

Exposure to B can vary based on dose and duration and may be acute (less than 14 days), intermediate (15–364 days), or chronic (365 days or more). Exposure routes include inhalation, oral intake, and dermal absorption, leading to a range of health effects such as systemic, immunological, neurological, reproductive, developmental, genotoxic, carcinogenic effects, and in severe cases, death [51, 53, 54]. In the air, B concentrations average 2.10 × 10^−5^ mg m^3^, with variations from > 5.10^−7^ to 9.10^−5^ mg m^3^, and its half‐life depends on particle size and atmospheric conditions [53]. In water, B typically has an average concentration of 0.1 mg•L^−1^, with fluctuations between < 1 and 3 mg•L^−1^. It can undergo various chemical reactions, including precipitation, polymerization, and acid–base reactions and, when hydrolyzed, forms boric acid (H_3_BO_3_) and the monovalent ion B(OH)_4_. In highly concentrated solutions with high pH, B may form complex molecules such as diborate [B_2_O(OH)_6_^2−^], triborate [B_3_O_3_(OH)_4_^−^], tetraborate [B_4_O_5_(OH)_4_^2−^], and pentaborate [B_5_O_6_(OH)_4_^−^] [51, 53]. Boron's adsorption–desorption in soils is influenced by factors such as pH, moisture, salinity, soil texture, and the chemical composition of the soil, including aluminum (Al) and iron (Fe) content. The average concentration of B in surface soil is 26 mg kg^−1^ [53].

Cu is a crucial micronutrient for plants, essential for processes like photosynthesis, respiration, and growth [55]. In humans, Cu is vital for a healthy diet, with an average daily intake of 1–2 mg/day. Copper deficiency can lead to anemia, weakened immunity, and insulin production issues [50]. Cu exposure depends on dose, duration, and the route of exposure—acute and intermediate exposure (0.02 mg Cu/kg/day), inhalation, or dermal absorption—can lead to liver, plasma, lung, kidney, ocular, and dermal problems [42, 56]. In water, Cu can either dissolve or bind to organic and inorganic particles, influenced by temperature, pH, and solubility [55]. The maximum permissible Cu levels in drinking water are 1.3 mg•L^−1^ for primary and 1.0 mg•L^−1^ for secondary drinking water (EPA), 2.0 mg•L^−1^ by the World Health Organization (WHO), and 1.0 mg•L^−1^ in bottled water (FDA) [42]. In soil, Cu adsorption depends on pH, organic matter content, metal oxyhydroxides, surface charge, and interactions with microorganisms [42].

Ni is a highly toxic non‐essential metal, even at low concentrations. It is persistent in the environment and bioaccumulates in animals. Depending on exposure routes—such as inhalation, oral intake, or dermal absorption—and the duration, Ni can cause a variety of health issues, including weight gain, bone and tooth damage (including gangrene of the extremities), and hematological, gastrointestinal, cardiovascular, respiratory, reproductive, neurological, immunological, endocrine, ocular, renal, hepatic, musculoskeletal problems, cancer, and death [50, 57]. Ni absorption, distribution, and elimination are influenced by factors like the physical form of the material, particle size (e.g., nanoparticles), and metal release [58]. Once ingested, Ni is eliminated from the body within approximately 28 h [59]. According to ATSDR [57], Ni concentrations in various environmental compartments include outdoor air (3–21.3 ng m^−3^), indoor air (2.79–23.7 ng m^−3^), groundwater (0–18.2 μg L^−1^), surface water (0–6.39 μg L^−1^), drinking water (0.55–25 μg L^−1^), soils (19.8–185 mg kg^−1^), and sediments (6–530 μg g^−1^).

Given the significant environmental and health impacts of organic and inorganic pollutants found in well water and cassava processing effluents, it is essential to analyze physical–chemical parameters and trace elements to assess water quality, safety, and ensure proper disposal practices.

### 3.4 Multivariate Analysis

Principal component analysis (PCA) was applied to the physicochemical parameters determined in the effluent lagoon and wellwater samples to identify patterns of variation and possible clustering between the samples.

The biplot obtained (Figure 4) demonstrates that the first principal component (Dim 1) explained virtually all of the data variability (100%), reflecting the overall difference between the two samples analyzed.

**FIGURE 4:**
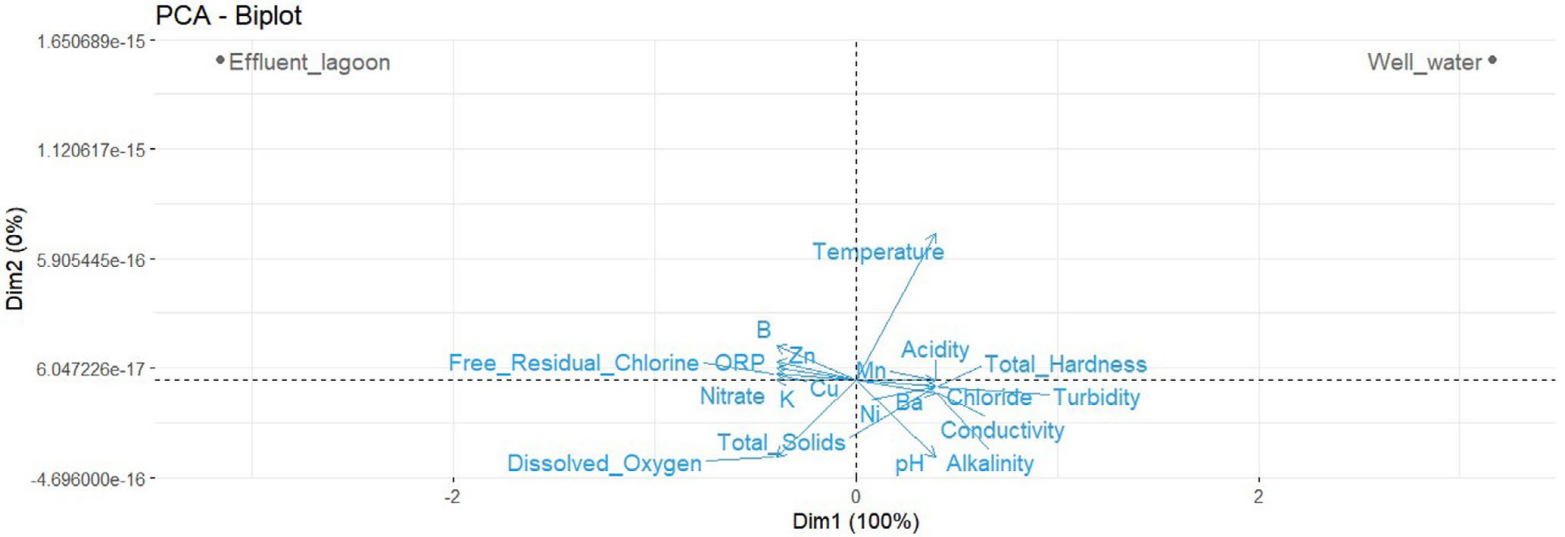
Biplot graphic of PCA. Relationship between samples, physicochemical parameters and mineral contents.

The separation observed along the axis of the first component indicates that the effluent lagoon and wellwater samples have distinct physicochemical profiles. The variables associated with temperature contributed most to the formation of Dim 1, being located close to the wellwater sample in the graph. This behavior suggests that temperature, dissolved oxygen, and pH are the main parameters responsible for distinguishing the two samples, presenting relatively higher variabilities in this matrix. This result is confirmed in Figure 5, that illustrates the contribution of variables to multivariate analysis.

**FIGURE 5:**
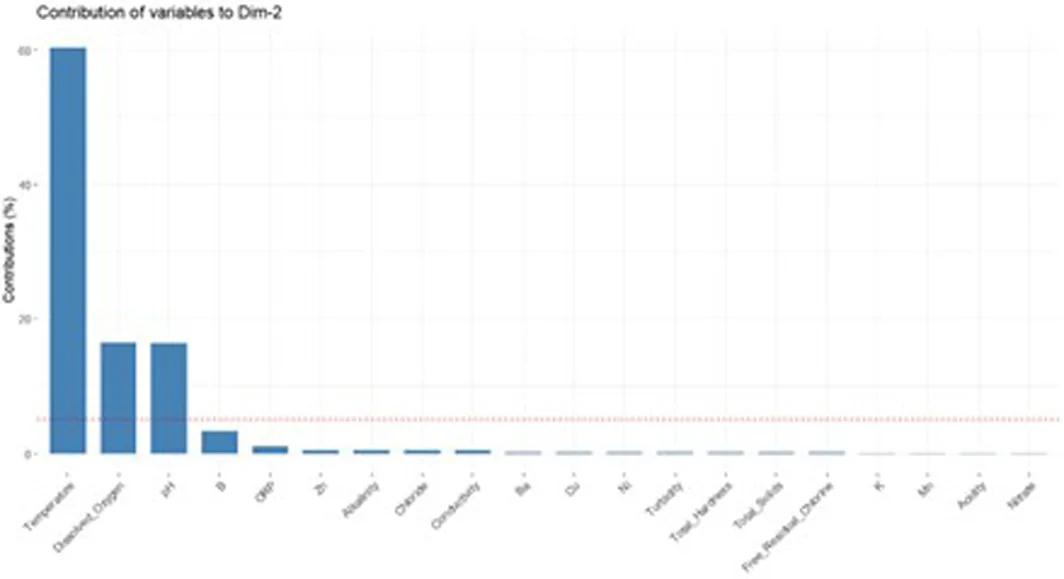
Contribution of variables to multivariate analysis.

On the other hand, parameters such as conductivity, turbidity, dissolved oxygen, and alkalinity were closer to the center of the biplot, indicating a smaller or similar contribution between the samples.

The lack of overlap between sampling points shows that, although the dataset consists of only two samples, there are significant differences in the ionic balance and physicochemical characteristics of each matrix.

Overall, the PCA confirmed the distinction between well water and lagoon effluent, suggesting that specific physicochemical processes (such as temperature variation and ionic composition) influence the properties of these samples.

## 4 Conclusion

This study highlights significant environmental and health implications associated with cassava processing effluents and the quality of artesian well water in the rural area of Bragança, PA. While no total or free cyanide was detected in the artesian well water, other parameters such as boron and copper exceeded the maximum limits set by Brazilian legislation, indicating potential risks to both human and environmental health. In the effluents, elevated levels of total and free cyanide, as well as manganese and nickel above the permissible limits, were detected. These findings underscore the need for actions to mitigate the environmental impacts of this activity. Overall, the study emphasizes the importance of implementing more sustainable practices for waste management, treatment, and monitoring in cassava processing, in order to protect both environmental and public health in the local communities.

## Author Contributions

A.C.M.: investigation, methods, analysis, discussion, writing – review and editing, tables, figures. M.G.N.: methods, analysis and discussion. L.P.S.: methods and analysis. T.M.S.: methods and analysis. E.N.M.J.: methods and analysis. H.A.D.F.: analysis, discussion, writing – review and editing, tables, figures and graphics. C.M.S.B.: analysis, graphics and discussion. K.G.F.D.: validation and supervision. S.F.P.P.: validation and supervision. I.C.C.B.: validation and supervision. B.J.M.C.: graphical abstract and English translation. R.S.P.: validation and supervision. N.M.P.A.: validation and supervision. A.S.L.: validation, supervision and financing acquisition.

## Funding

This study was funded by CAPES through Notice No. 13/2020 – Graduate Development Program (PDPG ‐ AMAZÔNIA LEGAL), linked to the Project Consolidation of Science in the areas of Food and Health in the Legal Amazon (Process No.: 88887.619858/2021‐00).

## Disclosure

This review article does not contain any studies with human or animal participants performed by any authors.

## Ethics Statement

Ethical approval was granted in accordance with the Certificate of Regularity of Access to Genetic Heritage and Associated Traditional Knowledge, registration no. AAA8C32, dated 05.06.2023, from the Genetic Heritage Management Council, Ministry of the Environment (MMA), Brazil.

## Consent

The authors have nothing to report.

## Conflicts of Interest

The authors declare no conflicts of interest.

## Data Availability

The data that support the findings of this study are available from the corresponding author upon reasonable request.
